# Smartphone-Based 3D Surface Imaging for Breast Anthropometry, Symmetry Assessment, and Volumetry: A Structured Narrative Review

**DOI:** 10.3390/jcm15166154

**Published:** 2026-08-07

**Authors:** Mateusz Mazurek, Zygmunt Domagała, Rafał Matkowski

**Affiliations:** 1 Clinical and Dissecting Anatomy Students’ Scientific Club, Wroclaw Medical University, 50-368 Wrocław, Poland; 2 Division of Anatomy, Department of Human Morphology and Embryology, Wroclaw Medical University, 50-368 Wrocław, Poland; zygmunt.domagala@umw.edu.pl; 3 Walbrzych Branch, Wroclaw Medical University, 58-300 Wrocław, Poland; 4 Department of Oncology, Wroclaw Medical University, 50-367 Wrocław, Poland; rafal.matkowski@umw.edu.pl; 5 Lower Silesian Oncology, Pulmonology and Hematology Center, 53-413 Wrocław, Poland

**Keywords:** smartphone-based 3D imaging, breast volumetry, breast anthropometry, breast surgery, breast measurement

## Abstract

**Background**: Objective breast assessment is relevant in aesthetic, reconstructive, and oncoplastic breast surgery. Smartphones and tablets are widely available devices that may offer an accessible means for three-dimensional (3D) breast surface imaging, with potential relevance for preoperative assessment, surgical planning, and outcome evaluation. However, the available evidence is heterogeneous, and the validity of different devices, applications, and workflows remains unclear. This narrative review aimed to summarize the evidence on smartphone-based 3D surface imaging. **Methods**: A structured literature search was conducted in PubMed, Embase, Web of Science, and EBSCOhost. Only original studies that used smartphone- or tablet-based breast assessment were included. Data extraction included devices, software, population/material, outcomes, comparators, validation metrics, and limitations. Reporting completeness was assessed using an adapted GRRAS-based appraisal. **Results**: A total of 9 studies representing approximately 7 independent datasets were included. Only two studies assessed volumetry as one of the primary aims. The evidence base was small and heterogeneous. Most studies were patient-based, while the remaining studies used phantom or model-based designs. Feasibility was demonstrated for selected device–application–workflow combinations, predominantly involving iPhones and the 3D Scanner App, but these findings were not generalizable to smartphone-based 3D breast imaging as a whole. Evidence was more favorable for selected linear anthropometric measurements than for volumetry. Key limitations included breast ptosis, inframammary fold (IMF) geometry, posterior boundary definition, software, and operator dependence. Three of the included reports had potentially overlapping cohorts. Due to substantial heterogeneity in devices, applications, comparators, outcomes, and validation metrics, meta-analysis was not performed. **Conclusions**: Current evidence is limited to a small number of heterogeneous, predominantly single-center datasets and supports only workflow-specific feasibility for selected anthropometric and surface-based measurements. These findings should not be generalized across smartphone devices, applications, or reconstruction workflows. Breast volumetry remains insufficiently validated and is supported by only two non-comparable studies. Future studies should provide more detailed reporting that focuses on objective characteristics of the workflow and outcomes, including predefined analyses of agreement and measurement error.

## 1. Introduction

Objective and reproducible assessment of breast morphology is relevant in aesthetic, reconstructive, and oncoplastic breast surgery, because surgical planning and postoperative outcome evaluation depend not only on subjective visual judgement, but also on quantifiable anatomical parameters [1]. Important parameters include breast volume, symmetry, nipple–areola complex position, inframammary fold (IMF) position, breast width, projection, and surface shape [1,2]. Breast volumetry might be a helpful tool aiding the choice of implants or flap planning [3]. Quantitative assessment might complement existing methods of subjective visual assessment.

Existing measurement methods vary in accuracy, accessibility, cost, and clinical practicality [2,3]. Breast morphology is 3D, so simple linear measurements or two-dimensional photographs cannot fully describe breast shape, surface, or volume [2,3]. Available methods include manual anthropometry, standard clinical photography, medical imaging, and commercial 3D scanning systems. Tape or caliper measurements are cheap and accessible, but can provide only selected linear measurements [2]. However, they are operator-dependent because landmark identification and measurement techniques may vary between examiners. Standard two-dimensional photography is useful for documentation, but it does not capture true 3D breast geometry. Therefore, it has limited value for breast volumetry and surface-based measurements [1,2]. Medical imaging methods, especially magnetic resonance imaging (MRI), can support breast volume assessment [3]. However, these methods are costly, not always easily available, and are usually performed for diagnostic indications rather than for routine aesthetic assessment [1,3]. Most medical imaging-based breast volume assessments are performed in non-upright positions, which may limit their ability to represent upright external breast morphology relevant to aesthetic assessment. Commercial 3D surface imaging systems, such as Vectra, 3dMD, or Artec, can capture breast surface morphology. They can support linear measurements, symmetry assessment, surface comparison, and breast volume estimation. However, these systems are expensive, less accessible, and require dedicated hardware and software [1].

For this reason, there is growing interest in more accessible 3D assessment methods, including smartphone-based imaging [3,4,5]. Advantages of smartphones include low cost, portability, wide availability, and ease of integration into clinical workflows [4,5]. Modern smartphones may use depth-sensing technologies, such as light detection and ranging (LiDAR) or TrueDepth cameras, or application-based photogrammetric or hybrid reconstruction workflows to create 3D surface models [4,6,7,8]. However, smartphone-based breast assessment is not a single method. It includes different devices, sensors, applications, scanning protocols, reconstruction algorithms, and post-processing steps. Because of this, evidence from one smartphone device, app, or protocol cannot be automatically generalized to smartphone-based breast assessment as a whole.

A reconstructed 3D model may look usable, but that does not prove its accuracy, agreement with established methods, reproducibility, repeatability, or clinical usefulness [9,10]. Linear anthropometry and volumetry impose different technical and anatomical requirements. Linear anthropometry is methodologically less demanding than volumetry, as volumetry requires a sufficiently complete mesh, breast segmentation, scale control, and standardized extraction [1,4]. 3D surface imaging of the breast also presents anatomical challenges, including IMF, ptosis, concave or hidden regions, and uncertainty in posterior breast boundary definition [4,11]. There are also workflow-related challenges, such as different software, segmentation procedures, post-processing, and operator dependence [5,8]. The aforementioned factors make it necessary to evaluate not only feasibility, but also reference methods, validation metrics, and error sources.

Current evidence on smartphone-based breast assessment is still limited and heterogeneous. Some studies compare smartphone-derived 3D models with commercial scanning systems [4,12,13], whereas others use manual measurements [5,6], MRI [11], or phantom reference volumes [8,14] as comparators. The available studies also differ in measured outcomes, including anthropometric distances [5], surface agreement [12], symmetry [7], and breast volume [11]. Therefore, it remains unclear which clinical applications are already supported by evidence and which still require further validation [4,6,12].

This narrative review aimed to synthesize current knowledge on smartphone-based breast assessment, with a focus on 3D surface imaging, anthropometry, symmetry assessment, and volumetry. It summarizes devices, software workflows, comparator methods, validation metrics, and reported limitations. The review focuses on how these methods are used, how accurate they are, what causes measurement error, and which areas require further study.

## 2. Materials and Methods

### 2.1. Design and Aim

This study was designed as a structured narrative review based on the predefined, multi-database search, eligibility criteria, and structured extraction. We have conducted a structured narrative review, meaning a narrative review with systematic elements in search, screening, and extraction, but without some measures required for systematic or scoping reviews, e.g., double screening or extraction. The choice of such methodology was determined by time and specialized personnel constraints. A meta-analysis was not planned because substantial heterogeneity was expected among the original studies in devices, software, acquisition protocols, comparators, and outcome measures. Evidence synthesis was therefore undertaken narratively. The Scale for the Assessment of Narrative Review Articles checklist (SANRA) was used to structure the manuscript [15].

This structured narrative review aimed to assess the evidence on smartphone-based methods for breast anthropometry and volumetry. Therefore, primary outcomes were volume, anthropometry, symmetry, and surface agreement. Secondary domains included comparator methods, such as MRI or manual measurements, validation metrics, and reported limitations. We also searched for reported limitations, determinants of measurement error, and evidence gaps in smartphone-based breast assessment techniques.

No prospective protocol registration was performed.

### 2.2. Search Strategy

We searched PubMed, Embase, Web of Science, and EBSCOhost. Additional hand searching was performed using the websites and recent contents of the Journal of Plastic, Reconstructive, and Aesthetic Surgery; Plastic and Reconstructive Surgery; and Journal of Clinical Medicine. Titles and abstracts identified through these searches were assessed using the same eligibility criteria as the database records. Preprints and gray literature were not systematically searched. The literature search was performed on 28 May 2026. The search queries were built around three concept blocks: device-related terms, breast-related terms, and measurement-related terms. The “Peer-reviewed journals” filter was used in EBSCOhost; no other filters were used. No language or publication date restrictions were applied. Original search queries for each database are available in Supplementary Materials.

### 2.3. Eligibility Criteria

The following eligibility criteria were used for study selection. To be included in the review, a study had to:

1. Use a smartphone as the acquisition device or central measurement tool;
2. Assess the breast, chest/breast surface, breast morphology, breast symmetry, breast anthropometry, or breast volume;
3. Use 2D images, 3D photogrammetry, LiDAR, structured mobile scanning, or smartphone-compatible 3D reconstruction;
4. Report any quantitative or methodological outcome;
5. Be original research studies, technical notes, validation studies, feasibility studies, or phantom/model studies.

The following studies were excluded:

1. Studies using only dedicated commercial 3D scanners without smartphone acquisition;
2. Studies using standard clinical photography without measurement or 3D/quantitative analysis;
3. Reviews, editorials, letters without original data;
4. Non-breast body surface scanning studies or studies oriented only on specific parts of the breast (e.g., nipple or nipple-areola complex).

### 2.4. Study Selection

All identified records were imported into Zotero 9.0.4. All duplicates were removed using the Zoplicate Build 5.0.7 add-on. Titles and abstracts were screened for eligibility. Full-text assessment was then conducted for potentially eligible studies. Additional hand searching was conducted using Google Search and selected journals relevant to breast surgery and aesthetic/reconstructive surgery. Only one author performed the screening.

### 2.5. Data and Synthesis

Data were extracted into a predefined extraction sheet by one author. Study identification, characteristics, design, population, context, outcome measures, key findings, and limitations were extracted. The full data extraction table is available in Supplementary Materials. No data imputation or second-reviewer verification was performed. The primary interest of this review was the feasibility of smartphone-based 3D breast surface imaging. Data on anthropometric measurements, volumetry, symmetry outcomes, surface agreement, validation references, and method limitations were extracted.

Thematic narrative synthesis was performed in the main domains. We collectively analyzed study characteristics, reporting quality, feasibility, anthropometry, surface agreement, volumetry, comparators, error sources, and evidence gaps. Different study designs, outcomes, and analysis methods in the primary studies did not allow for meta-analysis.

Moreover, three studies included possibly overlapping designs and cohorts. Those studies were included as separate publications due to different outcome measures presented in each of the studies. However, the results of those studies could not be pooled due to possible cohort overlap.

### 2.6. Adapted GRRAS-Based Reporting-Completeness Appraisal

Reporting completeness was assessed using an adapted approach based on the 15-item Guidelines for Reporting Reliability and Agreement Studies (GRRAS) checklist [10]. GRRAS was selected because most included studies investigated measurement agreement, reliability, repeatability, measurement error, or validation of smartphone-based breast assessment workflows. Some included publications were feasibility or clinical application studies rather than formal agreement studies; the GRRAS framework was considered relevant for assessing whether the measurement process, raters, statistical analyses, uncertainty, and practical interpretation were reported transparently.

All 15 original GRRAS items were retained and grouped according to the original sections: Title/Abstract, Introduction, Methods, Results, Discussion, and Auxiliary material. The adaptation consisted of assigning each item an ordinal reporting-completeness score: 0 = not reported or insufficiently reported, 1 = partially reported, and 2 = clearly and reproducibly reported.

Two reviewers independently assessed each publication. Pre-consensus agreement was calculated as percentage agreement across all study–item ratings, and discrepancies were resolved through discussion. The maximum score was 30 points per publication. These scores describe reporting completeness and methodological transparency only; they should not be interpreted as formal risk-of-bias assessments, measures of external validity, evidence of clinical validity, or a ranking of overall study quality.

### 2.7. Presentation of Data

Data were presented in tables, including characteristics of included studies, workflow characteristics, and anthropometric or volumetric agreement outcomes. Narrative synthesis was conducted.

## 3. Results

### 3.1. Study Characteristics

**Figure 1:**
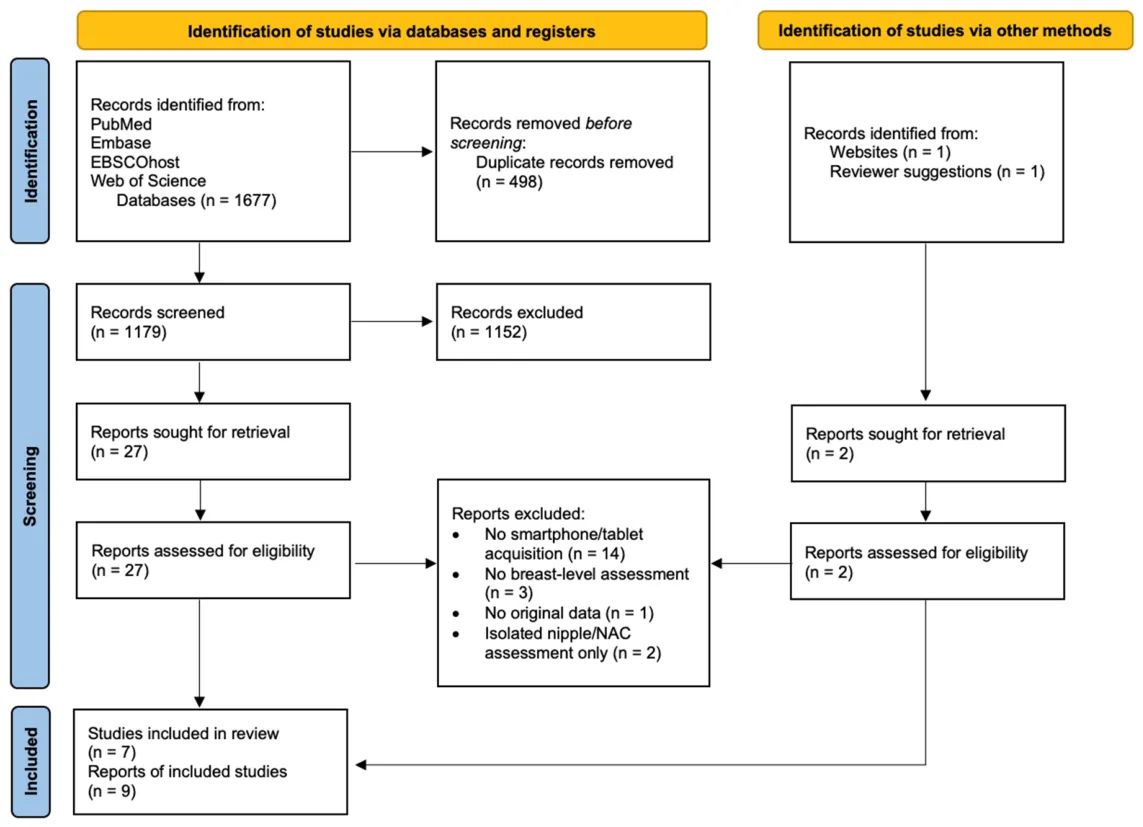
A flow diagram showing the study identification and selection process. The diagram was adapted from the PRISMA 2020 flowchart [16].

The database search identified 1677 records. After duplicate removal, 1179 records were screened. Twenty-seven full-text reports were assessed for eligibility. Eight reports were included from database searching, and one additional report was identified through hand searching, resulting in nine included publications. The full study selection process is presented in Figure 1. No records were excluded based on language.

Complete characteristics of included studies are presented in Table 1. The evidence base is small and heterogeneous. Most studies originated from European research groups, with two studies from the United States of America and the Republic of Korea. The majority of studies were patient-based, although phantom-based studies were also included. Most studies assessed linear anthropometry, symmetry, and surface agreement. Only a small number of studies assessed breast volume.

Several included publications were not fully independent. Chrobot et al. 2025a, Chrobot et al. 2025b, and Hartmann et al. 2025 originated from the same Regensburg-based research group and used closely related smartphone-based surface imaging workflows [7,12,13]. Participant-level overlap could not be confirmed from the published reports. The corresponding authors were not contacted because the timeline did not allow sufficient time for external clarification. Therefore, based on authorship, institutional setting, recruitment period, population characteristics, and closely related acquisition and analysis workflows, those reports were treated as related publications with probable workflow or cohort overlap. Therefore, the evidence base consisted of nine publications but seven fully independent datasets.

### 3.2. Reporting Quality Appraisal

Outcomes of reporting quality appraisal are presented in Table 2. GRRAS checklist appraisal yielded 135 items rated by two independent reviewers with agreement in 112 instances (percentage agreement 83%). Discrepancies were resolved by consensus.

Final consensus scores ranged from 18 to 28 out of 30. Highest reporting-completeness scores were observed in studies by Kyriazidis et al. (28 points), Botti et al. (26 points), and Chrobot et al. 2025a (26 points) [13]. Lowest scores were observed in studies by Han et al. (18 points), Hartmann et al. (20 points), and Dijkman et al. (23 points). In general, most studies presented higher scores for introductory rationale and relevance. Greater variability was found in methods, results, and auxiliary material. Higher scores should be interpreted as more complete reporting of information, not as formal risk-of-bias ratings or proof of clinical validity. The included publications differed in their primary aims; lower scores in agreement-specific domains may partly reflect study design rather than deficient conduct.

### 3.3. Smartphone-Based 3D Breast Surface Imaging Feasibility

**Figure 2:**
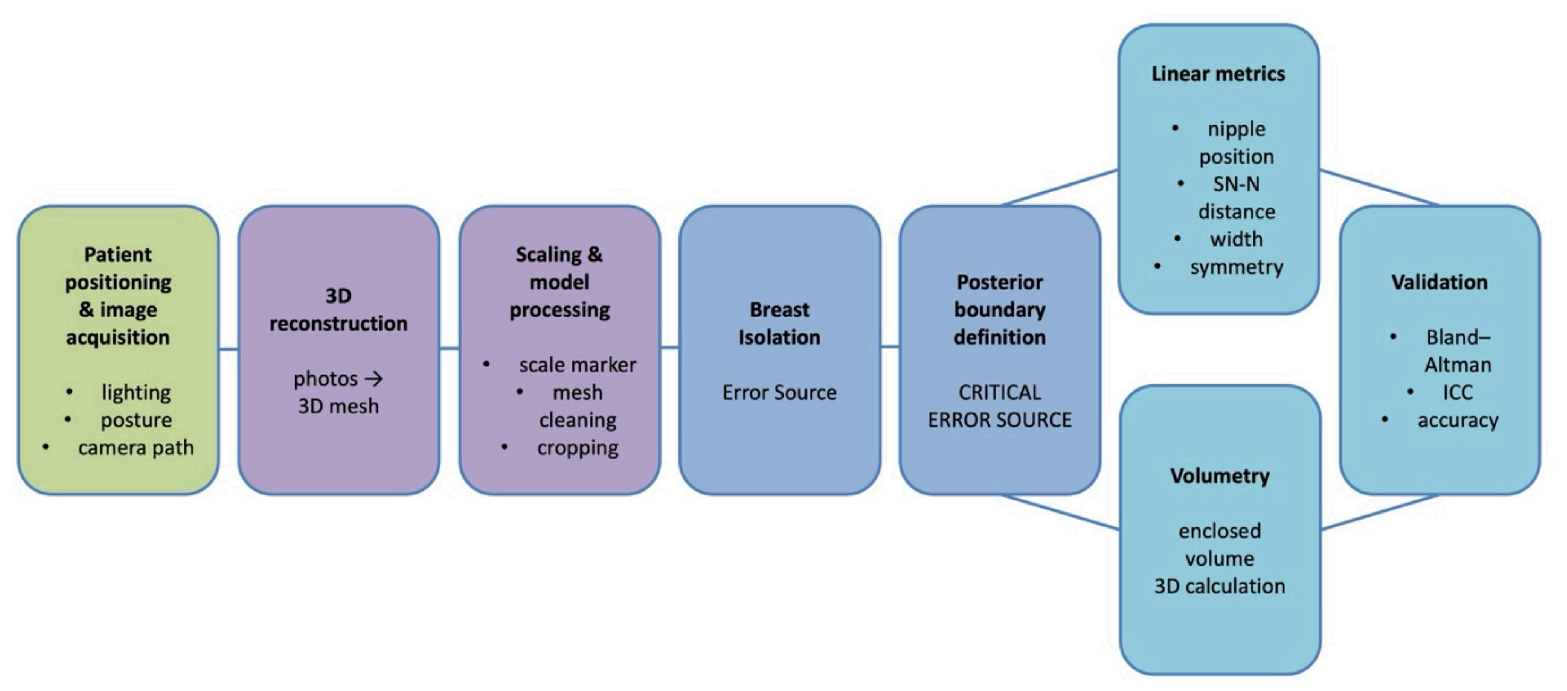
Conceptual workflow of smartphone-based 3D breast assessment. Standardized image acquisition is followed by photogrammetric reconstruction, model processing, breast isolation, posterior boundary definition, measurement extraction, and validation. Anthropometric and volumetric outputs are generated from the processed 3D model. Sources of error may occur at each stage of the workflow.

Across clinical and phantom-based studies, smartphone-based 3D breast imaging was generally feasible; an overview of the workflow is presented in Figure 2. Workflow complexity, model quality control, software dependence, and operator technique remain major constraints. The studies generally used different generations of iPhone and heterogeneous software workflows, including LiDAR-based, TrueDepth-based, and photogrammetric approaches. Feasibility characteristics are summarized in Table 3.

Although smartphone-based workflows generally generate analyzable 3D surface models, reporting remains inconsistent. Reconstruction success, failed scans, model exclusion rates, acquisition time, and post-processing time were not uniformly reported. Several studies required additional software for model modification, indicating that smartphone-based 3D surface model acquisition is not yet a standardized measurement workflow.

### 3.4. Anthropometric and Symmetry Assessment

Studies were generally focused on anthropometry, symmetry, and surface agreement, not volumetry, as shown in Table 4. Outcomes included landmark-to-landmark distance, breast width, sternal notch to nipple distance (SN-N), nipple to inframammary fold distance (N-IMF), nipple to midline distance (N-M), nipple to nipple distance (N-N), midclavicle to nipple distance (MC-N), IMF length, symmetry index, and surface-to-surface deviation.

Agreement between smartphone-based and manual measurements was generally favorable for selected visible landmarks, including SN-N, N-M, and N-N, whereas N-IMF showed higher relative error [5,6]. These estimates should be interpreted cautiously because confidence intervals were not consistently reported, particularly for TEM and rTEM outcomes. Manual tape measurement is a routinely used practical tool, and smartphone-based measurement yielded similar outcomes for most landmarks, except those related to the IMF.

Commercial 3D imaging systems, such as Vectra H2, Vectra XT, or Artec LEO, were used as comparator systems in several studies [4,12,13]. Agreement between these scanners and smartphones was generally good. However, two studies using Vectra H2 appear to derive from the same sample. Smartphones also showed acceptable agreement with Vectra XT and Artec LEO systems for surface measurements [14].

Smartphone-based 3D surface imaging was also used to assess symmetry in a comparison of immediate and delayed-immediate breast reconstruction [7]. An important consideration is that this study comes from the Regensburg cohort and, therefore, its patients, methods, and outcomes may overlap with those of two other articles [12,13].

Smartphone-based 3D surface imaging is generally good for easily accessible landmarks, such as the sternal notch, nipple, midline, midclavicle, exposed breast poles, and relatively flat areas. However, lower visibility of certain areas of the breast, especially in the case of high-grade ptosis or IMF, causes problems.

### 3.5. Smartphone-Based Breast Volumetry

Evidence on smartphone-based volumetry is limited. Only two studies measured volumetric outcomes [11,14].

Only one study compared smartphone-based volumetry with MRI-derived breast volume as a clinical imaging reference [11]. Smartphone-based 3D surface imaging can be used for volumetry, but it showed a negative bias compared with MRI. The magnitude and precision of this bias differed between observers and breast sides, and the original study reported uncertainty using concordance estimates and Bland–Altman analysis. The authors suggested that this bias may be operator- and anatomy-dependent. Larger breasts generally had higher absolute errors. This practical investigation highlights important limitations, especially problems with defining the posterior breast boundary, which is not visible in surface imaging. The IMF may also lead to defects in reconstructed models. Manual segmentation and boundary definition require training and standardization; therefore, the method is operator-dependent.

One phantom-based study also assessed 3D surface imaging volumetry against a known reference volume [14]. Smartphone-based volumetry appeared to be less accurate than commercially available 3D scanning systems; however, the reported error was interpreted by the authors as acceptable in the controlled phantom setting.

Studies related to anthropometric and surface area measurements do not directly prove the possibility of volumetry. However, their agreement with established methods and good accuracy results are promising for future assessment of volumetric capabilities of 3D surface imaging.

### 3.6. Reference Methods and Validation Quality

Reference methods and validation approaches differed substantially between studies, as shown in Table 4. MRI, commercial 3D systems, manual techniques, and known volume phantoms were used.

Each comparator method serves a different purpose. Volumetric comparison with MRI provides data for preoperative in vivo assessment in clinical practice [11]. Commercial scanning systems are validated tools for breast surgery planning and outcomes assessment [4,12,13,14]. Manual linear measurement is common in daily practice [5,6]. Phantom-based measurements allow assessment of the technical qualities of the method while eliminating biological sources of random error.

Study outcomes are highly heterogeneous and are reported using TEM and rTEM. Intraclass correlation, Bland–Altman analysis, and the concordance correlation coefficient were used to assess agreement between methods, measurements, and assessors. Descriptive statistics such as RMSE, mean absolute error (MAE), surface deviation, and mean difference were also used.

Meta-analysis of those studies was not undertaken as they present differing outcomes, comparators, units of analysis, metrics, and populations. Moreover, Regensburg-originated studies present overlapping methods, outcomes, and cohorts, which could negatively impact unbiased assessment. Therefore, the evidence was synthesized narratively.

### 3.7. Error Sources and Methodological Limitations

Across studies, the main limitation was the ability to obtain and analyze the model in a standardized manner. Determinants of error may be characterized as anatomical, technical, and operator-dependent.

The breast is anatomically complex and variable in projection, ptosis, IMF geometry, skin-to-skin contact, posterior boundary definition, and position-dependent shape. For linear measurements, this can lead to errors in anatomical landmark distances, especially those involving the inferior pole of the breast, due to ptosis and the IMF. For volumetry, the main problem is the posterior breast boundary, as volume measurement requires a closed or closable surface with predefined boundary rules.

Technical and software-related errors can be divided into five types. First, sensor-related limitations reflect the accuracy of different 3D surface imaging techniques. Second, mesh quality can be affected by holes, artifacts, blurred regions, incomplete surfaces, poor textures, and noise; such errors may make it impossible to close the model for volumetric measurement without excessive structural modification, which could directly influence volumetric outcomes. Third, scaling is another possible source of error. Many studies included alignment, scaling, cropping, or mesh editing before measurements. Ideally, scale should be controlled at acquisition or verified before measurement. However, if scaling is necessary, it should follow a standardized workflow. Fourth, software-dependent errors stem from app algorithms. Finally, another error source is the use of multiple software programs within a single workflow, which was common in the available studies.

Operator-dependent error sources may arise during acquisition or analysis. Acquisition-related decisions include scanning speed, distance, trajectory, number of images, patient movement, lighting, arm position, and capture of high-risk regions such as the IMF. Analysis-related decisions include manual landmarking, segmentation, mesh closure, model acceptability assessment, and correction. Most importantly, the operator analyzing the model is responsible for approximating the posterior breast boundary.

For anthropometry, many of the abovementioned sources of errors may be irrelevant. Volumetry requires a closed mesh, scaling, breast region definition, posterior breast boundary approximation, and a strict, standardized workflow for reproducibility of the method.

Smartphones can create 3D surface models of the breast, yet those models are not always acceptable for anthropometric or volumetric measurements, especially if they may have clinical implications. The most important problems for volumetry are posterior breast boundary approximation, mesh closure, and operator-dependent variability.

### 3.8. Gap for Future Studies

The available literature suggests feasibility, but the evidence remains poorly standardized. Existing studies have small samples, include overlapping cohorts, and have not been replicated. They use heterogeneous comparators and different outcome measures. Repeatability and reproducibility are not commonly reported. Reconstruction success rates are also rarely reported. No standardization has yet been introduced to the field, and future work should focus on this gap. Future studies should move toward standardized, reproducible, reference-compared workflows for smartphone-based breast anthropometry and volumetry.

## 4. Discussion

We identified a small body of recently published articles. The included studies generally supported the feasibility of smartphone-based workflows for generating analyzable images or 3D surface models. However, the strength of evidence was not uniform across different types of measurements. Anthropometric linear measurements, surface agreement with commercial 3D systems, and symmetry-oriented assessment had the strongest evidence [4,5,6,12,13]. In contrast, the evidence for volumetric assessment remained limited and less robust. The literature is heterogeneous in devices, software, acquisition protocols, comparators, and validation metrics [4,8,11,12,13,14]. The interpretation of the evidence is limited by partial overlap between included publications. Three reports from the same Regensburg-based group used closely related workflows and likely overlapping clinical populations. Although these studies provide useful information on smartphone-based anthropometry, symmetry assessment, and surface-model comparison, they should not be interpreted as independent replications of the same findings. Consequently, conclusions regarding the feasibility and reliability of these applications should be weighted according to the smaller number of independent datasets rather than the number of publications alone. Treating the three related Regensburg publications as a single dataset did not change the overall narrative interpretation, but reduced the apparent degree of independent replication supporting smartphone-based anthropometry, symmetry assessment, and surface comparison. The conclusions regarding volumetry remained unchanged because these publications did not provide independent volumetric validation.

Smartphone-based 3D surface imaging appeared feasible across the included studies. Generated 3D models were suitable for post-processing and analysis. However, in most cases, feasibility was inferred from successful analysis rather than formal reporting of technical parameters. In most studies, reconstruction success, failed scans, exclusion rates, acquisition time, post-processing time, and model quality criteria were not systematically reported. Measurement validity requires agreement with a reference standard, reliability testing, a standardized workflow for acquisition and post-processing, and transparent reporting of failures [9,10]. The reporting-completeness appraisal showed that most studies did not report feasibility as a predefined or measurable endpoint. Only one study provided a transparent discussion of handling poor-quality scans [5]. One limitation concerns rapid hardware and software iteration. The included studies used different smartphone generations, sensors, applications, algorithms, and post-processing workflows. Therefore, smartphone-based 3D imaging should not be interpreted as a single, stable measuring workflow, but rather as a developing measurement technology. Available findings obtained with one workflow or device may not be directly generalized to another workflow. Detailed reporting of technical characteristics in future studies is crucial for workflow repeatability and possible clinical application.

Available evidence is more favorable for linear anthropometry and symmetry assessment than for volumetry, as presented in Table 5. Most studies reported linear parameters and compared them with manual measurement techniques or commercial 3D scanning systems. The agreement between smartphone-based imaging and established methods is generally good for selected outcomes. Better performance was observed for easily visible and anatomically stable landmarks and landmark-to-landmark distances, such as SN–N, N–M, N–N, and breast width. In contrast, measurements related to the lower breast contour and the IMF were more error-prone. Current evidence is based on methodological papers, rather than clinical validation and standardization. Symmetry indices require further assessment of agreement with patient- and surgeon-reported outcomes, long-term postoperative assessment, and relevance in clinical decision-making. Therefore, smartphone-based imaging may be useful for anthropometric measurements; however, it should not be generalized to all kinds of measurements or volumetry.

Compared with anthropometry, evidence on smartphone-based volumetry remains limited. Only two studies directly assessed volume, one in a clinical setting and one in phantoms. These two settings answer different validation questions and should not be interpreted as equivalent evidence. The clinically relevant comparison with MRI-derived breast volume as a reference method showed that smartphone-derived estimates may systematically differ from MRI-derived estimates [11]. Only one available phantom-based study is useful, as it provides known reference volumes, but it does not reproduce breast ptosis, IMF anatomy, skin folds, or tissue deformities [14]. Phantom-based validation also does not fully reproduce one of the most important sources of volumetric error: posterior breast boundary definition and closure. Volumetry is more technically demanding than linear measurements because it requires a sufficiently complete mesh, proper scaling, breast segmentation, posterior boundary definition, and standardized volume extraction rules. Therefore, linear measurements do not prove volume accuracy. A recent borderline study on smartphone-video-based nipple volume measurement using Gaussian splatting was not included because it focused exclusively on isolated nipple volumetry rather than whole-breast surface assessment or breast-level volumetry. Nevertheless, it illustrates the rapid expansion of smartphone-compatible 3D reconstruction methods and reinforces the need for endpoint-specific validation of each device–software–workflow combination [17].

**Figure 3:**
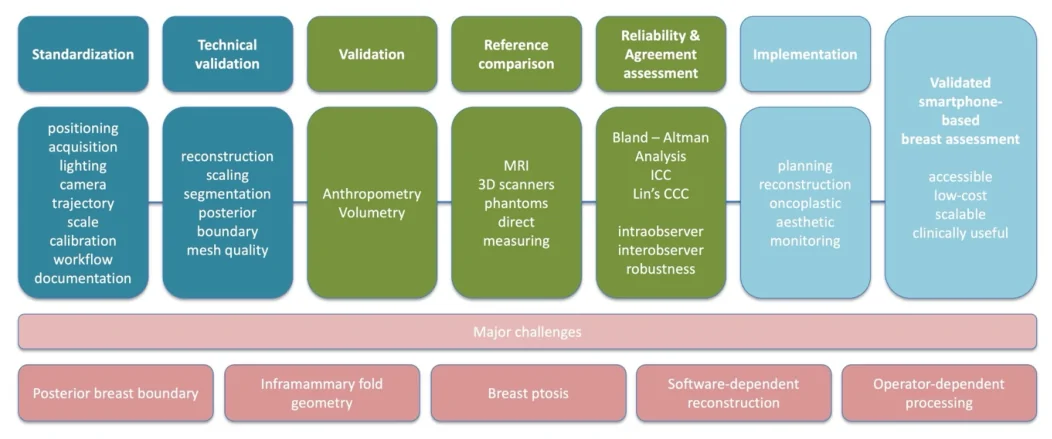
Research roadmap for validation of smartphone-based breast assessment. Proposed framework outlining the progression from methodological standardization and technical validation to measurement validation, reference comparison, reliability assessment, and clinical implementation. Major unresolved challenges include posterior boundary definition, inframammary fold geometry, breast ptosis, software-dependent reconstruction, and operator-dependent processing.

Existing studies used different comparator methods, as listed in Table 3. Different comparators answer different research questions. MRI was used as a reference method for volumetric agreement assessment. Commercial scanners were used for surface geometry assessment [4,12,13,14]. Manual measurements using tape measures and calipers were used as comparator methods for anthropometric measurements. Phantom reference studies assessed technical accuracy [8,14], while some studies were conducted in a clinical setting, but for application, not validation [7]. Studies used different validation metrics, including Lin’s CCC, ICC, Bland–Altman analysis, TEM/rTEM, RMSE, MAE, surface-to-surface deviation, and between-group clinical comparisons. The GRRAS-informed appraisal showed that reporting completeness varied across the included studies. Therefore, favorable agreement or reliability results should be interpreted in relation to how completely the underlying measurement workflow was reported. Meta-analysis was considered inappropriate because of reporting inconsistencies and substantial heterogeneity in outcomes, comparators, populations, and validation metrics. Smartphone-based breast assessment seems to be a promising method that could positively influence preoperative planning in oncoplastic and aesthetic breast surgery. However, it should not be evaluated as one general method. Rather, validation should depend on the workflow specific to the intended application. Specific suggestions are mentioned in Figure 3.

This structured narrative review addresses a narrow but clinically and methodologically relevant topic. The search was structured and used multiple databases with a wide array of indexed journals. Eligibility criteria and extracted data were predefined. The review presents the results in separate outcome domains, with tables presenting extracted data. However, a crucial limitation of this article is that this was a structured narrative review, not a systematic review with meta-analysis, even though the search and extraction were structured. Deviations from a systematic protocol include the lack of a pre-registered protocol, independent screening and extraction, and broad criteria that are more fitting for a structured narrative review than for a systematic review. Both screening and extraction were performed by one author, and only GRRAS-informed assessment was conducted by two independent reviewers. Patient-level overlap between the related Regensburg publications could not be confirmed from the published reports. The corresponding authors were not contacted to verify individual participant overlap, recruitment overlap, or shared patient identifiers. Therefore, overlap was inferred from the research group, setting, recruitment periods, population descriptions, and methodological workflow, and this uncertainty should be considered when interpreting the synthesis. Quantitative analysis was not possible because of the heterogeneity of the reports. The assessed evidence base was small, but this stems from the narrow scope of the review. Overall, current evidence supports the feasibility of smartphone-based breast surface imaging for selected anthropometric and surface-based applications, but it does not yet support routine volumetric use without further workflow-specific validation.

## 5. Conclusions

This review identified 9 publications representing 7 independent datasets. Current evidence supports workflow-specific feasibility for selected smartphone-based anthropometric and surface-assessment applications. The evidence was dominated by iPhone-based workflows, frequently using the 3D Scanner App, and several validation studies relied on Vectra systems as the principal comparator. Evidence is stronger for selected anthropometric and symmetry outcomes than for volumetry. Breast volumetry requires further validation. Interpretation is limited by the small and heterogeneous evidence base. Some of the currently available reports present overlapping methods, cohorts, and results. Future studies should aim for standardized workflows and reference-based validation with agreement assessment based on the Bland–Altman analysis. Independent, multicenter validation across different devices, applications, operators, comparators, and patient populations is required before routine clinical implementation can be supported.

## Figures and Tables

**Table 1:** Characteristics of included studies. * Chrobot et al. 2025a, Chrobot et al. 2025b, and Hartmann et al. 2025 originated from the same Regensburg research group and used closely related or potentially overlapping workflows and cohorts [7,12,13].

Study	Country	Population/Material	Sample Size	Smartphone	Acquisition Method	Comparator
Behrens et al. 2024 [11]	Germany	Female patients undergoing routine/standard-of-care breast MRI	22	iPhone 11 Pro Max	Smartphone-based 3D body surface scanning using integrated optical 3D/TrueDepth sensor and 3D scanner app	3.0 Tesla MRI [MAGNETOM Vida, Siemens] with dedicated 16-channel bilateral breast coil. Non-enhanced T1w 3D Dixon in-phase images used. Semiautomatic threshold-based workflow in Syngo.via/MM Reading, performed by board-certified radiologist. MRI volume segmentation required approximately 5 min per breast.
Botti et al. 2025 [8]	Belgium	Silicone-based female torso phantom/mannequin with silicone breasts, fiducial markers, and a Lego block for scale calibration.	36	iPad Pro 3	Mobile 3D scanning applications; five workflows used the iPad built-in camera/depth-estimation approach, and one used the Structure Sensor external depth sensor.	Linear distances between marker pairs measured using digital calipers by two independent operators; mean manual value treated as ground truth.
Chrobot et al. 2025a [13]	Germany	Women after breast reconstruction surgery; smartphone-based and Vectra H2 3D surface models generated for each participant.	40 *	iPhone 15 Pro	Smartphone-based 3D surface imaging using iPhone 15 Pro LiDAR sensor in conjunction with photogrammetry/photo mode.	Vectra H2 used as validated reference surface imaging tool; iPhone and Vectra surface models superimposed and compared using CloudCompare/Vectra Analysis Module (VAM).
Chrobot et al. 2025b [12]	Germany	Patients after breast reconstruction surgery	40 *	iPhone 15 Pro	Smartphone-based 3D surface imaging using 3D Scanner App Photo Mode; captures image series every 0.8 s from different angles; advanced camera features including LiDAR/camera/TrueDepth available	Vectra H2 portable stereophotogrammetry system with VAM software, considered established/gold-standard mobile 3D surface imaging comparator for breast assessment by the authors
Dijkman et al. 2024 [14]	The Netherlands	Female mannequin with clay breast prostheses of known volumes; reference prostheses of 75 cc, 100 cc, and 125 cc; baseline mannequin without prostheses used as control.	20	iPhone XR	3D scanning using iPhone XR TrueDepth camera with Heges app; compared with Vectra XT photogrammetry and Artec LEO VCSEL scanner.	Clay prostheses of 75, 100, and 125 cc had reference volume determined with Archimedes’ principle. Volume differences estimated by each 3D method were compared against expected prosthesis volumes. Baseline model without prosthesis was used for subtraction.
Han et al. 2023 [6]	Republic of Korea	Human participants undergoing breast geometric measurement with iPhone-based LiDAR 3D scanning and tapeline measurement.	46	iPhone 12 Pro	LiDAR-based 3D scanning using iPhone; RGB point cloud acquisition and 3D model reconstruction.	Tapeline measurements used as ground truth/reference for distances and circumference.
Hartmann et al. 2025 [7]	Germany	Women after breast reconstruction surgery using deep inferior epigastric perforator (DIEP) flap	26 *	iPhone 15 Pro	Smartphone-based 3D surface imaging using 3D Scanner App photo mode; LiDAR sensor combined with photogrammetry	Study used smartphone-derived 3D surface models only; groups compared by reconstruction timing rather than against Vectra/MRI/manual measurements
Kyriazidis et al. 2025 [5]	Belgium	Female patients undergoing breast procedures; clinical patient cohort.	25	iPhone 15 Pro	Smartphone LiDAR-based 3D scanning using the 3D Scanner App; circular scan around patient.	Traditional tape measurements performed by two surgeons; LiDAR scanning also performed by surgeons. Observers were blinded to each other’s results; measurements were performed twice. Tape measurement was chosen as the reference standard due to widespread clinical use.
Rudy et al. 2024 [4]	USA	Postoperative patients after breast reconstructive surgery by autologous free tissue transfer.	10	iPhone X	3D smartphone scanning using front-facing iPhone X 3D scanner with ScandyPro; rotating front face of iPhone around patient.	Vectra H2 scans were automatically processed by Canfield stereophotogrammetric stitching software; no additional postprocessing. iPhone models were imported into VAM and compared with Vectra using iterative closest point (ICP) alignment, colormap analysis and surface distances.

**Table 2:** GRRAS-informed reporting-quality appraisal of the included studies [10]. Scores are descriptive and aimed at assessment of reporting completeness. Comparison between studies of different designs should be done carefully. Scores are summarized by GRRAS section, with item-level ratings assigned as 0 = not reported or insufficiently reported, 1 = partially reported, and 2 = clearly and reproducibly reported. The total score ranges from 0 to 30. Agreement indicates pre-consensus item-level agreement between the two independent reviewers; all disagreements were resolved through consensus discussion. Color coding was used for visual interpretation only and does not represent statistical significance or formal quality assessment. Dark green indicates the highest reporting-completeness scores, light green intermediate scores, yellow moderate scores, and red the lowest reporting-completeness scores.

Study	Title/ Abstract /2	Introduction /8	Methods /10	Results /6	Discussion /2	Auxiliary /2	Total /30	Agreement (*n*/15)	Agreement %	Disagreements (*n*)
Behrens et al. 2024 [11]	2	7	7	5	2	1	24	13/15	86.7%	2
Botti et al. 2025 [8]	2	7	9	5	2	1	26	11/15	73.3%	4
Chrobot et al. 2025a [13]	2	7	9	5	2	1	26	12/15	80.0%	3
Chrobot et al. 2025b [12]	2	7	7	5	2	1	24	12/15	80.0%	3
Dijkman et al. 2024 [14]	2	6	7	4	2	2	23	13/15	86.7%	2
Han et al. 2023 [6]	2	5	5	3	2	1	18	10/15	66.7%	5
Hartmann et al. 2025 [7]	0	6	7	4	2	1	20	12/15	80.0%	3
Kyriazidis et al. 2025 [5]	2	8	9	6	2	1	28	15/15	100.0%	0
Rudy et al. 2024 [4]	2	7	7	5	2	1	24	14/15	93.3%	1

**Table 3:** Acquisition settings and protocol details. Chrobot et al. 2025a, Chrobot et al. 2025b, and Hartmann et al. 2025 originated from the same Regensburg research group and used closely related or potentially overlapping workflows and cohorts [7,12,13].

Study	Setting	Device and Software	Position and Acquisition	Post-Processing Workflow	Feasibility Notes
Behrens et al. 2024 [11]	Clinical	iPhone 11 Pro Max; 3d Scanner App™; MeshLab; Meshmixer 3.5	Upright standing position; arms elevated 90°; smartphone mounted on a manually moved rail; scan trajectory across the anterior thorax and both breasts	PLY export; MeshLab for file reduction/rough repair; Meshmixer for processing, defect closure, segmentation, and volume calculation	Feasible clinical acquisition, but workflow required dedicated positioning setup and multi-software post-processing.
Botti et al. 2025 [8]	Phantom	iPad Pro 3; Structure Sensor; 3D Scanner App; Heges; Polycam; SureScan; Kiri Engine; Blender	Silicone breast phantom under controlled lighting; two operators; three scans per application	Digital distances measured on reconstructed meshes in Blender and compared with caliper measurements	Demonstrated feasibility across several mobile applications, but performance was strongly software-dependent.
Chrobot et al. 2025a [13]	Clinical	iPhone 15 Pro; 3D Scanner App V2.1.2; CloudCompare ICP; MeshLab; Vectra Analysis Module	Upright position; arms at 45° using a stick; Vectra and smartphone scans acquired sequentially; anatomical stickers used	OBJ export; cropping; CloudCompare ICP alignment/scaling; MeshLab processing; VAM surface-to-surface/landmark analysis	Feasible in a clinical breast reconstruction surgery (BRS) cohort, but dependent on sequential scanning, landmarking, alignment, and external software.
Chrobot et al. 2025b [12]	Clinical	iPhone 15 Pro; 3D Scanner App; CloudCompare; MeshLab	Upright position; arms at 45° using a stick; smartphone photo-mode scans acquired after Vectra H2	Cropping, scaling/alignment in CloudCompare, normals and Screened Poisson processing in MeshLab, measurements in VAM	Feasible, but preprocessing was laborious and required several software steps.
Dijkman et al. 2024 [14]	Phantom	iPhone XR; Heges; GOM-inspect	Mannequin with reference prostheses; 20 repeated measurements per volume condition; iPhone mounted on rotating arm	STL export; GOM-inspect for aligned, cut, and filled models; Vectra software also used for Vectra XT volumes	Controlled feasibility for volume-change assessment; iPhone was lowest-cost but less accurate than dedicated systems.
Han et al. 2023 [6]	Clinical	iPhone 12 Pro; Innoscan	Sitting position; hands overhead; operator moved 360° around participant at approximately 50 cm	In-app RGB 3D model reconstruction with artifact denoising/body-segment detection; in-app distance and circumference measurements	Feasible integrated app-based workflow, but IMF-related measurements were problematic.
Hartmann et al. 2025 [7]	Clinical	iPhone 15 Pro; 3D Scanner App V2.1.2; CloudCompare; Vectra Analysis Module	Upright position; 30–40 cm distance; uniform lighting; landmarks applied; scans repeated if motion artifacts or failed scans occurred	OBJ export; cropping in CloudCompare; anthropometric measurements and symmetry index calculation in VAM/Excel	Clinical application of smartphone imaging; likely related to the Regensburg BRS dataset; feasibility supported but not independent validation.
Kyriazidis et al. 2025 [5]	Clinical	iPhone 15 Pro; 3D Scanner App	Upright position; arms behind back; 800–1000 lux diffuse lighting; nonreflective background; 1–1.5 m device distance; three revolutions per scan	In-app mesh visualization and measurements; full scan repeated if holes, blur, or misalignment were present	Strongest reporting of acquisition quality control; workflow emphasizes operator technique and real-time mesh assessment.
Rudy et al. 2024 [4]	Clinical	iPhone X, ScandyPro; Meshlab; Blender; Vectra Analysis Module	Upright position; arms abducted 45°; iPhone rotated around patient; additional lower-angle pass to capture IMF	OBJ export; Blender/MeshLab cropping and hole filling; VAM rigid alignment, colormap, and landmark measurements	Feasible and rapid acquisition, but analysis required external post-processing and plastic-surgery-specific software was lacking.

**Table 4:** Main outcomes, metrics, and comparators. Measures of precision are reported where available; where confidence intervals or equivalent uncertainty measures were not provided in the original publication, this is stated explicitly. Detailed outcomes are available in the original articles and Supplementary Materials Extraction Table.

Study	Measurements/Assessed Features	Comparator	Precision/ Uncertainty	Main Validation Metrics	Key Findings	Main Limitation Relevant to Interpretation
Behrens et al. 2024 [11]	Breast volume extraction from smartphone-derived 3D surface models	MRI-derived breast volume	CCC 0.87–0.98; Bland–Altman analysis reported.	Concordance correlation coefficient (CCC), Bland–Altman analysis, mean difference, interobserver comparison	Smartphone-derived volumes generally underestimated MRI volumes; interobserver CCC was high, but agreement with MRI varied by observer	Primarily volumetric rather than anthropometric; posterior boundary, IMF defects, breast size, and upright-vs-prone mismatch affect interpretation
Botti et al. 2025 [8]	Linear distances between fiducial markers on silicone breast phantom	Manual caliper measurements	Mean errors reported with SD; no CIs.	Mean absolute error, standard deviation, Kruskal–Wallis test, Dunn post hoc test, operator variability analysis	SureScan, Structure Sensor, Heges, 3D Scanner App, and Kiri showed lower errors than Polycam; Polycam had highest error and variability	Phantom-based only; no clinical tissue deformation, ptosis, or patient movement; not direct clinical anthropometry
Chrobot et al. 2025a [13]	Fourteen landmark-to-landmark surface deviations after superimposition of smartphone- and Vectra-based models	Vectra H2	Mean deviation 2.997 ± 1.897 mm; 95% LoA reported	Absolute error, paired tests, Bland–Altman analysis, intraclass correlation coefficient (ICC)	Average landmark-to-landmark deviation was 2.997 ± 1.897 mm; reliability was good to excellent; 13/14 measurements showed moderate to reliable agreement	Same/related Regensburg cohort; sequential scanning and alignment may introduce error; not independent from companion publications
Chrobot et al. 2025b [12]	Fourteen breast anthropometric measurements, including SN-N, LBP-N, UBP-N, Xi-N, LaBP-N, breast width, and IMF length	Vectra H2	ICC 0.963–0.998; 95% LoA within ±2 mm	ICC, Bland–Altman analysis, paired tests	ICC ranged from 0.963 to 0.998; no significant differences in 11/14 measurements; 95% limits of agreement (LoA) remained within ±2 mm	Laborious post-processing; no independent cohort relative to companion Regensburg publications; workflow depends on external software
Dijkman et al. 2024 [14]	Surface deviation analysis of baseline models; breast volume differences using reference prostheses	Known prosthesis volume; Vectra XT and Artec LEO compared with iPhone XR	RMSE reported; no CIs	Relative mean difference, root mean square error (RMSE), surface deviation analysis	iPhone XR had larger relative mean differences and RMSE than Vectra XT and Artec LEO; baseline surface deviation vs. Vectra XT approximately +0.33 mm	Mannequin-based; iPhone setup less accurate than dedicated systems; not direct patient-based anthropometry
Han et al. 2023 [6]	MC-N, SN-N, N-IMF, N-N distance, nipple-level and IMF-level circumferences	Manual tapeline measurements	TEM/rTEM reported; no CIs	Technical error of measurement (TEM), relative TEM (rTEM), *p*-values	Good rTEM for most measurements: 2.99–5.19%; poor rTEM for N-IMF distance: 16.64% right and 19.76% left	IMF-related measurements were inaccurate, likely due to ptosis and difficulty capturing the inframammary region
Hartmann et al. 2025 [7]	Fourteen anthropometric measurements and symmetry index after immediate vs. delayed-immediate DIEP flap reconstruction	No external measurement reference; comparison between clinical groups	Effect sizes and *p*-values reported; CIs not consistently reported	Independent samples *t*-test, symmetry index, Cohen’s d	No significant difference in symmetry index between immediate and delayed-immediate reconstruction; 11/14 measurements did not differ significantly	Clinical application rather than validation; probable subset/related Regensburg dataset; SI not externally validated against perceptual or clinical outcomes
Kyriazidis et al. 2025 [5]	SN-N, N-M, N-IMF	Manual tapeline measurements	ICC 0.82–0.95 and rTEM reported; no CIs	TEM, rTEM, ICC, learning curve analysis	Very good rTEM for SN-N and N-M; poor rTEM for N-IMF: approximately 13%; interrater reliability excellent, ICC 0.92	IMF measurement remained unreliable; operator technique and scanning quality strongly influenced results
Rudy et al. 2024 [4]	SN-N, nipple-to-midchest, nipple-to-mid-IMF, IMF width; colormap surface comparison	Vectra H2	Mean discrepancy 0.909 ± 0.073 mm; 95% LoA −1.90 to 2.17 mm	Bland–Altman analysis, mean absolute discrepancy, surface RMSE	Mean absolute landmark discrepancy was 0.91 mm; colormap RMSE 1.53 mm; Bland–Altman bias 0.13 mm with LoA −1.90 to 2.17 mm	Small sample; no severe ptosis; post-processing required; volume estimation considered inaccurate due to hidden chest-wall geometry

**Table 5:** Evidence-readiness summary for smartphone-based 3D breast assessment applications.

Application Domain	Current Evidence Status	Main Validation Priority
Linear anthropometry	Promising for selected visible landmarks	Standardized landmark definitions and repeated validation across independent cohorts
Symmetry assessment	Promising but not independently replicated	Independent validation of symmetry metrics against clinical and patient-reported outcomes
Surface agreement	Promising in selected device–workflow combinations	Workflow-specific comparison with established commercial 3D systems
Breast volumetry	Insufficiently validated	MRI- or reference-volume-based agreement studies with standardized segmentation and boundary rules

## Data Availability

The original contributions presented in this study are included in the article. Further inquiries can be directed to the corresponding author.

## Supplementary Materials

The following supporting information can be downloaded at: https://www.mdpi.com/article/10.3390/jcm15166154/s1.

## Abbreviations

The following abbreviations are used in this manuscript:

3D	three-dimensional
BRS	breast reconstruction surgery
CCC	concordance correlation coefficient
DIEP	deep inferior epigastric perforator
ICC	intraclass correlation coefficient
ICP	iterative closest point
IMF	inframammary fold
LiDAR	light detection and ranging
LoA	limits of agreement
MAE	mean absolute error
MC–N	midclavicle-to-nipple distance
MRI	magnetic resonance imaging
N–IMF	nipple-to-inframammary fold distance
N–M	nipple-to-midline distance
N–N	nipple-to-nipple distance
RMSE	root mean square error
rTEM	relative technical error of measurement
SN–N	sternal notch-to-nipple distance
TEM	technical error of measurement
VAM	Vectra Analysis Module
