# Supplementary material for: Smartphone-Based 3D Surface Imaging for Breast Anthropometry, Symmetry Assessment, and Volumetry: A Structured Narrative Review
Source: J Clin Med. 2026 Aug 7;15(16):6154. doi: 10.3390/jcm15166154 (PMC13512946; doi:10.3390/jcm15166154)
Supplement: Supplementary file 1 [file jcm-15-06154-s001.zip › jcm-4398497-supplementary.pdf]

Pubmed
(smartphone OR "mobile phone" OR iPhone OR LiDAR OR photogrammetry) AND (breast OR breasts OR "breast surgery" OR "breast reconstruction" OR "breast volume") AND (volume OR volumetry OR anthropometry OR "surface imaging" OR "3D imaging" OR "three-dimensional" OR symmetry OR morphology OR measurement OR scanning)

Embase
('smartphone'/exp OR smartphone:ti,ab,kw OR 'mobile phone'/exp OR 'mobile phone':ti,ab,kw OR iphone:ti,ab,kw OR lidar:ti,ab,kw OR photogrammetry:ti,ab,kw)
AND
('breast'/exp OR breast:ti,ab,kw OR breasts:ti,ab,kw OR 'breast surgery':ti,ab,kw OR 'breast reconstruction'/exp OR 'breast reconstruction':ti,ab,kw OR 'breast volume':ti,ab,kw)
AND
('volume'/exp OR volume:ti,ab,kw OR volumetry:ti,ab,kw OR anthropometry:ti,ab,kw OR 'surface imaging':ti,ab,kw OR '3d imaging':ti,ab,kw OR 'three dimensional':ti,ab,kw OR 'three-dimensional':ti,ab,kw OR symmetry:ti,ab,kw OR morphology:ti,ab,kw OR measurement:ti,ab,kw OR scanning:ti,ab,kw)

Web of Science
TS=(
(smartphone OR "mobile phone" OR iPhone OR LiDAR OR photogrammetry)
AND
(breast OR breasts OR "breast surgery" OR "breast reconstruction" OR "breast volume")
AND
(volume OR volumetry OR anthropometry OR "surface imaging" OR "3D imaging" OR "three-dimensional" OR symmetry OR morphology OR measurement OR scanning)
)

Ebsco
(
(TI (smartphone* OR "mobile phone*" OR cellphone* OR "cell phone*" OR iPhone* OR iPad* OR LiDAR OR "light detection and ranging" OR photogrammetry)
OR AB (smartphone* OR "mobile phone*" OR cellphone* OR "cell phone*" OR iPhone* OR iPad* OR LiDAR OR "light detection and ranging" OR photogrammetry))
AND
(TI (breast* OR mammary OR mammaplasty OR mammoplasty OR "breast surgery" OR "breast reconstruction" OR "breast volume")
OR AB (breast* OR mammary OR mammaplasty OR mammoplasty OR "breast surgery" OR "breast reconstruction" OR "breast volume"))
AND
(TI (volume OR volumetry OR volumetric OR anthropometry OR anthropometric OR "surface imaging" OR "3D imaging" OR "3D scan*" OR "three dimensional" OR "three-dimensional" OR symmetry OR morphology OR measurement* OR scanning OR scan*)
OR AB (volume OR volumetry OR volumetric OR anthropometry OR anthropometric OR "surface imaging" OR "3D imaging" OR "3D scan*" OR "three dimensional" OR "three-dimensional" OR symmetry OR morphology OR measurement* OR scanning OR scan*))
)
Automatic filter: „peer-reviewed journals”
